# Extended phenotypic spectrum of benign yellow dot maculopathy

**DOI:** 10.1038/s41433-024-03590-4

**Published:** 2025-02-21

**Authors:** Peter Kiraly, Johannes Birtel, Ariel Y. Ong, Claire Ruan, M. Dominik Fischer, Peter Charbel Issa

**Affiliations:** 1 Oxford Eye Hospital, Oxford University Hospitals NHS Foundation Trust, Oxford, UK; 2 Nuffield Laboratory of Ophthalmology, Nuffield Department of Clinical Neurosciences, University of Oxford, Oxford, UK; 3 Department of Ophthalmology, University Medical Center Hamburg-Eppendorf, Hamburg, Germany; 4 Centre for Ophthalmology, University Hospital Tübingen, Tübingen, Germany; 5 Department of Ophthalmology, Technical University of Munich, School of Medicine and Health, TUM University Hospital, Munich, Germany

**Keywords:** Retinal diseases, Eye manifestations

## Abstract

**Background:**

To present the morphological and functional characteristics of individuals with benign yellow dot maculopathy (BYDM).

**Methods:**

Assessments included ocular examinations, best-corrected visual acuity (BCVA) testing, optical coherence tomography (OCT), blue-light fundus autofluorescence (BAF), and near-infrared autofluorescence (NIR-AF). First degree family members were also examined whenever available.

**Results:**

25 individuals with BYDM (15 females, 10 males) from 19 unrelated families with a median age at first presentation of 37 years (range, 4–54 years) were included in the study. The 19 index patients were referred for assessment of early-onset drusen (*n* = 10), macular dystrophy (*n* = 6), or an unrelated ocular condition (*n* = 3). Clinical examination of 15 first-degree family members of 8 probands revealed vertical transmission in 6 relatives. After excluding 6 patients with other ocular pathologies, BCVA was 20/25 or better in all patients. Fundoscopically, all patients had yellow dots in the macular area, extending to the vascular arcades in 19 and beyond in 11 individuals. Hyper-autofluorescent dots on BAF topographical matched the dots seen on fundoscopy, while hypo-autofluorescent dots were noted on NIR-AF. OCT revealed no abnormalities in 14 cases, but mild ellipsoid zone irregularities were observed in 11. No morphological or functional progression was noted in 15 individuals over an average follow-up period of 3.6 years.

**Conclusion:**

BYDM may present with a mild phenotype with yellow dots extending to the vascular arcades and beyond, suggesting it could be more common than previously reported. Recognizing this phenotype may reduce unnecessary investigations and follow-ups. Yellow dots show hypo-autofluorescence on NIR-AF and there is no morphological or functional progression.

## Introduction

Benign yellow dot maculopathy (BYDM) was first described in 2017 in a multi-center cohort of 36 individuals [1]. Since then, there has only been five further case reports or case series which together have reported 16 additional patients [2–8]. Consistent findings include bilateral yellow dots at the level of the retinal pigment epithelium (RPE) of the central retina, which are usually discrete and evenly distributed, although some may be confluent [1]. These dots show an increased signal on fundus autofluorescence (AF) imaging, and while optical coherence tomography (OCT) is unremarkable in the majority of patients, few may exhibit minimal irregularities at the interface between photoreceptors and the RPE [1]. Three cases of unilateral BYDM have been reported, although a slightly different distribution of the yellow dots could indicate a different etiology [4, 7–8]. Retinal function is usually normal, affected individuals are asymptomatic, and non-progression has been documented for up to 18 years [3]. Vertical transmission of BYDM may be observed in some family trees, but genetic associations have not yet been identified [1].

The significance of BYDM is that individuals with this condition are often diagnosed with early-onset drusen or a macular dystrophy, leading to unnecessary investigations, follow up examinations, and associated psychological burden. Awareness of this condition and accurate diagnosis at initial presentation can therefore avoid this.

Herein, we report multi-modal retinal imaging findings of 25 individuals with BYDM which indicate an extended phenotypic spectrum of this condition, and postulate a hypothesis on the structural basis of BYDM.

## Methods

### Subjects

In this retrospective study, all individuals were investigated at the Oxford Eye Hospital, Oxford University Hospitals NHS Foundation Trust, Oxford, UK and presented initially between 2016 and 2023. The study was registered as a clinical audit and was exempt from requiring formal ethics approval. All clinical investigations were performed as part of routine clinical care. This work adhered to the tenets of the Declaration of Helsinki, and all subjects provided informed consent.

Ophthalmic assessment was carried out according to standard departmental practice, including visual acuity measurements, fundus autofluorescence (AF) imaging using blue (B-AF; 488 nm, barrier filter at 500 nm; Spectralis HRA-OCT, Heidelberg Engineering, Heidelberg, Germany) and near-infrared (NIR-AF; excitation at 787 nm, barrier filter at 810 nm; HRA2, Heidelberg Engineering) excitation light. Spectral-domain optical coherence tomography (OCT) imaging (Spectralis HRA-OCT, Heidelberg Engineering) and fundus color photography (TRC-50DX, Topcon, Tokyo, Japan) were also performed.

The inclusion criteria comprised the presence of yellow small dots on fundoscopy, indicative of BYDM, and corresponding hyper-autofluorescence as detected with blue-light autofluorescence imaging. Whenever feasible, first-degree family members were also examined.

## Results

We identified 25 individuals with BYDM. Nineteen index patients were referred for a second opinion on suspected early-onset drusen (*n* = 10), macular dystrophy (*n* = 6) or for an unrelated ocular condition (*n* = 3; BYMD as incidental finding). Clinical examination of 15 first-degree family members of 8 index subjects identified the additional 6 individuals, which supported an autosomal dominant transmission (Table 1). In 3 out of 4 instances where both parents were investigated, one of the parents showed few yellow dots, indicating mild BYDM. Male-to-male transition was observed in one family.

**Table 1:** Characteristics of 25 individuals with benign yellow dot maculopathy (BYDM).

#	Sex	Age [years]	Follow-up [months]	BCVA [logMAR]		Distribution and extent of yellow dots	OCT changes related to yellow dots	Other visually relevant ocular pathology	Clinical examination of family members
				RE	LE				
1	M	8	27	0.1	0.1	diffuse, severe	none		both parents, 1 sibling
1-mother	F	45	12	0	0	few, temporal > nasal	none		
2	F	40	74	0	0	diffuse, severe	mild EZ irregularities		1 sister
2-sister	F	45	74	0	0	diffuse, moderate, temporal > nasal	mild EZ irregularities		
3	F	51	---	0	0	diffuse, moderate	none		1 daughter
4	M	25	71	0	0	diffuse, moderate	mild EZ irregularities		both parents, one sibling
4-mother	F	62	---	0.1	0	few	mild EZ irregularities		
5	F	37	52	0	0	central, moderate, mainly inferior	none		2 children
6	M	39	18	0	0	diffuse, moderate, temporal > nasal	none		
7	M	31	85	0.1	0	central, severe – additionally few diffuse	mild EZ irregularities		
8	F	38	6	0	0	diffuse, moderate. Denser nasal central	none		
9	M	25	---	0.3	0.3	central > diffuse, moderate	none	bilateral astigmatism	
10	M	24	---	0	0	central > diffuse, moderate	mild EZ irregularities		
11	F	53	78	0.4	0.48	diffuse, moderate, temporal > nasal	none	MacTel	
12	F	9	---	0	0	few	None		both parents
12-mother	F	39	---	0	0	few	none		
13	M	44	---	0	0	diffuse, moderate	none	pachychoroid / CSC	
14	F	38	---	0	0	diffuse, few, mainly temporal	none		
15	F	34	43	0	0	central, moderate	none		both parents
16	F	28	---	0	0	central, moderate, nasal > temporal, additionally few diffuse	mild EZ irregularities		
17	M	33	62	0	0	central, moderate, nasal > temporal	mild EZ irregularities		
18	F	52	---	0.78	0	diffuse, moderate, temporal > nasal	mild EZ irregularities	right amblyopia	
19	M	36	28	0.39	NA	central, moderate	mild EZ irregularities	autosomal dominant nanophthalmos associated with a mutation in TMEM98	1 daughter, 1 son
19-daughter	F	9	17	0.17	0.17	central, moderate	mild EZ irregularities		
19-son	M	4	9	NA	NA	central, few	none		

Family members are only included in the table if they presented changes compatible with the diagnosis of BYMD.

*BCVA* Best corrected visual acuity, *logMAR* Logarithm of the Minimum Angle of Resolution, *RE* right eye, *LE* left eye, *OCT* optical coherence tomography, *EZ* ellipsoid zone, *PED* pigment epithelial detachment, *CSC* central serous chorioretinopathy, *MacTel* macular telangiectasia type 2.

Median age at presentation was 37 years (range, 4–54 years) and 16 (59%) individuals were female. Mean [±SD] BCVA was logMAR 0.10 (±0.18) in the right eye and 0.05 (±0.12) in the left eye. Seven patients had reduced vision due to other ocular pathologies (Table 1) and after exclusion of their eyes, BCVA was logMAR 0.1 or better in all cases (mean ± SD BCVA: logMAR 0.01 ± 0.03 in right eyes and 0.00 ± 0.02 in left eyes). All individuals without additional ocular pathology were asymptomatic.

**Fig. 1:**
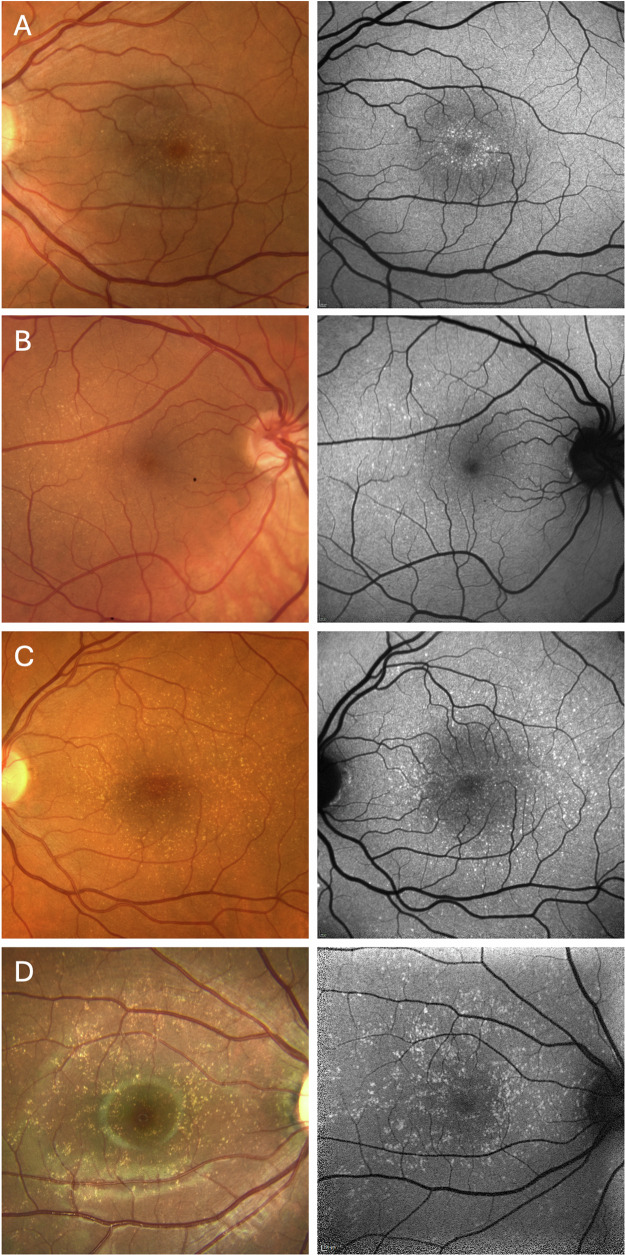
Extended spectrum of benign yellow dot maculopathy (BYDM) phenotypes. Patient with localized and clustered circular yellow dots next to the fovea (**A**); Patient with mild phenotype and yellow dots mostly temporal to the fovea (**B**); Patients with a broader yellow dots distribution extending to the vascular arcades and periphery (**C**, **D**). (**A**: #7, **B**: #11, **C**: #2, **D**: #1). Color fundus photo (left column),  blue autofluorescence (right column).

The yellow dots at the posterior pole showed an overall high symmetry between right and left eyes. The topographic distribution of yellow dots differed between patients: the lesions could be limited to a central oval area with approximate dimensions of 1 disc diameter vertically and 1.5 disc diameters horizontally (Fig. 1A), whereas others showed a more diffuse distribution at the posterior pole (Fig. 1B, C), in some cases extending beyond the temporal arcades (all patients presented in supplementary Figs. 1 and 2). No yellow dots were observed in the far periphery of the retina. The density of lesions also showed large variability, ranging from almost confluent accumulation to only few individual dots. The latter finding was often observed in family members and could have been interpreted as normal variation without the context of the index patients (e.g., #1-mother and #4-mother; supplementary Fig. 1). Density distribution was sometimes asymmetric, independent of the overall involved area, e.g., with denser yellow dots in the nasal central area (e.g., #16, Supplemental Fig. 2) or more dots in more eccentric areas temporally (Fig. 1B). Some eyes with mainly central yellow dots showed a few additional lesions in more eccentric areas, usually temporally. Overall, there was no indication of an increased area or density of yellow dots in older individuals as more extensive BYDM was observed in young individuals and very mild forms in older probands (e.g #1 and #1-mother).

**Fig. 2:**
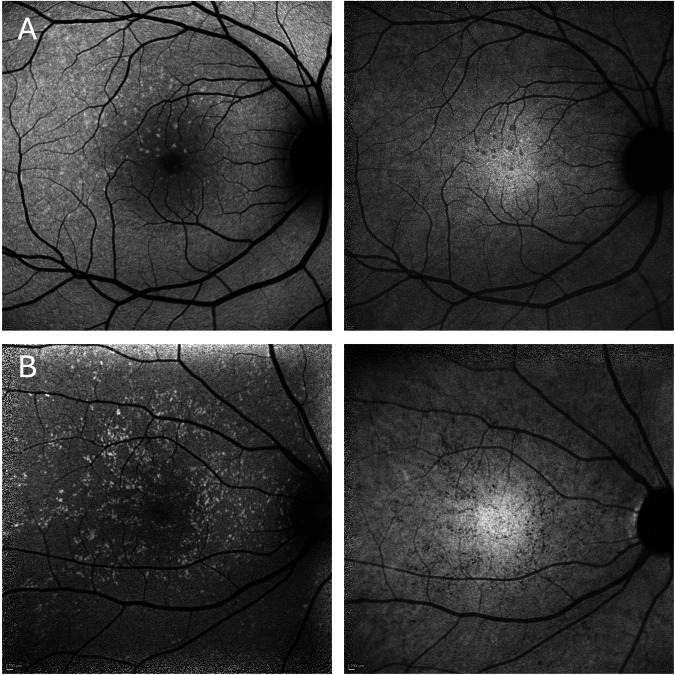
Blue autofluorescence and near infrared autofluorescence of benign yellow dot maculopathy (BYDM). Hyperautofluorescent dots on blue autofluorescence (left column) corresponding to hypoautofluorescent dots on near infrared autofluorescence (right column). (**A**: #15, **B**: #1).

**Fig. 3:**
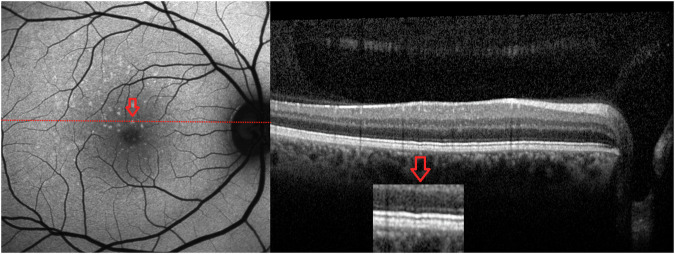
Optical coherence tomography (OCT) reveals mild ellipsoid zone irregularities in some patients (#15).

On blue AF (*n* = 25) and NIR-AF (*n* = 19) imaging, the yellow dots corresponded to an increased and reduced signal, respectively (Fig. 2). OCT was unremarkable in 14 patients and revealed very mild ellipsoid zone (EZ) irregularities in 11 patients (Fig. 3). Three patients showed OCT changes related to a macular co-pathology (# 11, 13, 19).

Follow-up data was available for 16 patients, with a mean [±SD] interval of 3.5 years [±2.3 years; range, 6 months – 7 years]. None of the patients without other ocular pathologies developed visual symptoms or showed changes in BCVA, yellow dots and other structural assessments.

## Discussion

Herein, we report the second largest cohort of individuals with BYDM to date. All individuals were seen at a single center, indicating a higher prevalence than expected based on the few previous reports in the literature. Moreover, we identified some family members of index patients with only few yellow dots which we interpreted as very mild presentation of BYDM. The prevalence of such mild manifestations is unknow and without the context of the index patient, it would have been unlikely for these patients to have been diagnosed with BYDM.

In this cohort, we confirm several key findings in individuals with BYDM. This includes a possible autosomal dominant inheritance, an increased short wavelength autofluorescence of the yellow dots, minimal or non-detectable changes on OCT imaging, a lack of visual function impairment and non-progression [1–8]. The latter, together with the observation that extensive BYDM may be present in children, indicates a developmental origin of BYDM, although formal confirmation would be required. We also confirm absence of similar lesions in the fundus periphery – a finding that may be used to distinguish BYDM from North Carolina macular dystrophy (NCMD) [9]. Nevertheless, distinction of these two entities may still be challenging in some cases: both conditions may present with a dominant pedigree and similar macular changes, although findings in BYDM would consistently be at the milder end of the spectrum observed in NCMD.

The topographic distribution of the yellow dots varied substantially in our cohort. Images shown in a previous report [1] indicate that most dots are concentrated in the macular area, sometimes with higher density in the nasal macular area. However, the same authors mentioned the rare observation of few dots outside the temporal vascular arcades.

We present cases with wider involvement of the posterior pole and suggest that BYDM may present as a spectrum with regards to distribution and density of lesions, although it remains possible that these cases represent other conditions that may produce a similar phenotype.

A new finding in this series is the reduced autofluorescence signal on NIR-AF imaging that corresponded to hyper-autofluorescence on SW-AF and yellow dots on fundoscopy. A possible explanation may be that the yellow dots in BYDM are caused by a lack of melanin in RPE cells. Lipofuscin and melanin are the two main pigments in RPE cells [10]. Lipofuscin, which is excited at short wavelengths of the visible spectrum (e.g., blue light), is visualized with SW-AF, while melanin is excited at longer wavelengths and may be visualized using NIR-AF imaging [10, 11]. Therefore, lack of melanin would be seen on NIR-AF as hypoautofluorescence [12], which is consistent with our findings in BYDM patients. At the same location, melanin would absorb less of the short wavelength blue excitation light and hence could enhance the visualization of lipofuscin, an orange-yellow autofluorescent material that may appear as yellowish dots on clinical examination. This hypothesis on the structural basis of the yellow dots would also account for the lack of significant changes on OCT images of BYDM patients. Alternative explanations include accumulation of a fluorophore visible on B-AF imaging, while another fluorophore that fluoresces on NIR-AF imaging decreases, or that the accumulating fluorophore absorbs NIR-light. However, this would imply dynamic changes of the yellow dots over time which has not yet been documented.

Most probands in our cohort were referred due to suspected early-onset drusen or macular dystrophy, both of which are potentially blinding conditions. Therefore, accurately identifying BYDM in everyday clinical practice is crucial to reduce the psychological burden associated with the progression and vision loss commonly seen in inherited retinal dystrophies. Additionally, correct diagnosis of BYDM can eliminate the need for genetic testing, second opinion referrals, and follow-up examinations, thereby reducing the burden on the healthcare system as well.

In conclusion, our study suggests that BYDM is non-progressive and may be more prevalent than previously reported, exhibiting an extended phenotypic spectrum and a mild phenotype in many patients. Multimodal imaging has led us to hypothesize that the yellow dots observed might represent focal areas lacking melanin in the RPE. The widespread recognition of BYDM is crucial in order to facilitate accurate diagnosis, thus alleviating the psychological burden for patients, as well as reducing unnecessary investigations and follow-up examinations.

## Summary

### What was known before

- Benign yellow dot maculopathy has recently been recognized as a specific entity.
- Patients are usually asymptomatic and retinal function is usually normal.
- Vertical transmission has been observed in families of some patients.

### What this study adds

- The prevalence of benign yellow dot maculopathy is possibly higher than previously assumed.
- The extend of retinal involvement is very variable, resulting in a wide spectrum of phenotypic presentation.
- The yellow dots appear dark on near-infrared autofluorescence imaging, possibly indicating a developmental focal lack of melanin in retinal pigment epithelial cells.

## Supplementary information

Supplementary Figure 1

Supplementary Figure 2

## Data Availability

Data generated or analysed during this study are included in this published article and its supplementary information files. More specific information is available from the corresponding author on reasonable request.
