# Supplementary figures and images for: Extended phenotypic spectrum of benign yellow dot maculopathy
Source: Eye (Lond). 2025 Feb 21;39(8):1547–52. doi: 10.1038/s41433-024-03590-4 (PMC12089353; doi:10.1038/s41433-024-03590-4)

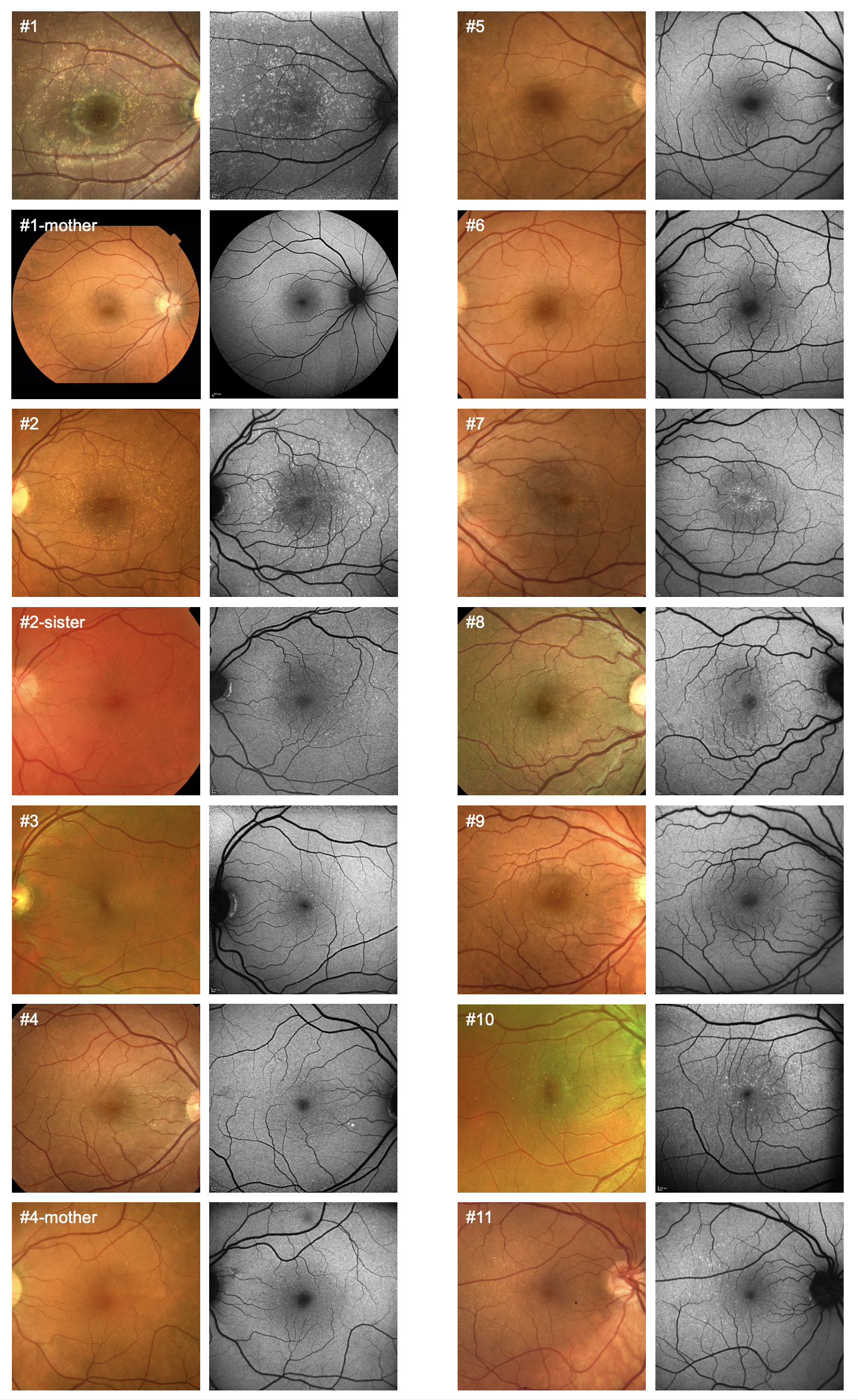

Supplement: Supplementary file 1 — Supplementary Figure 1 [file 41433_2024_3590_MOESM1_ESM.jpg]

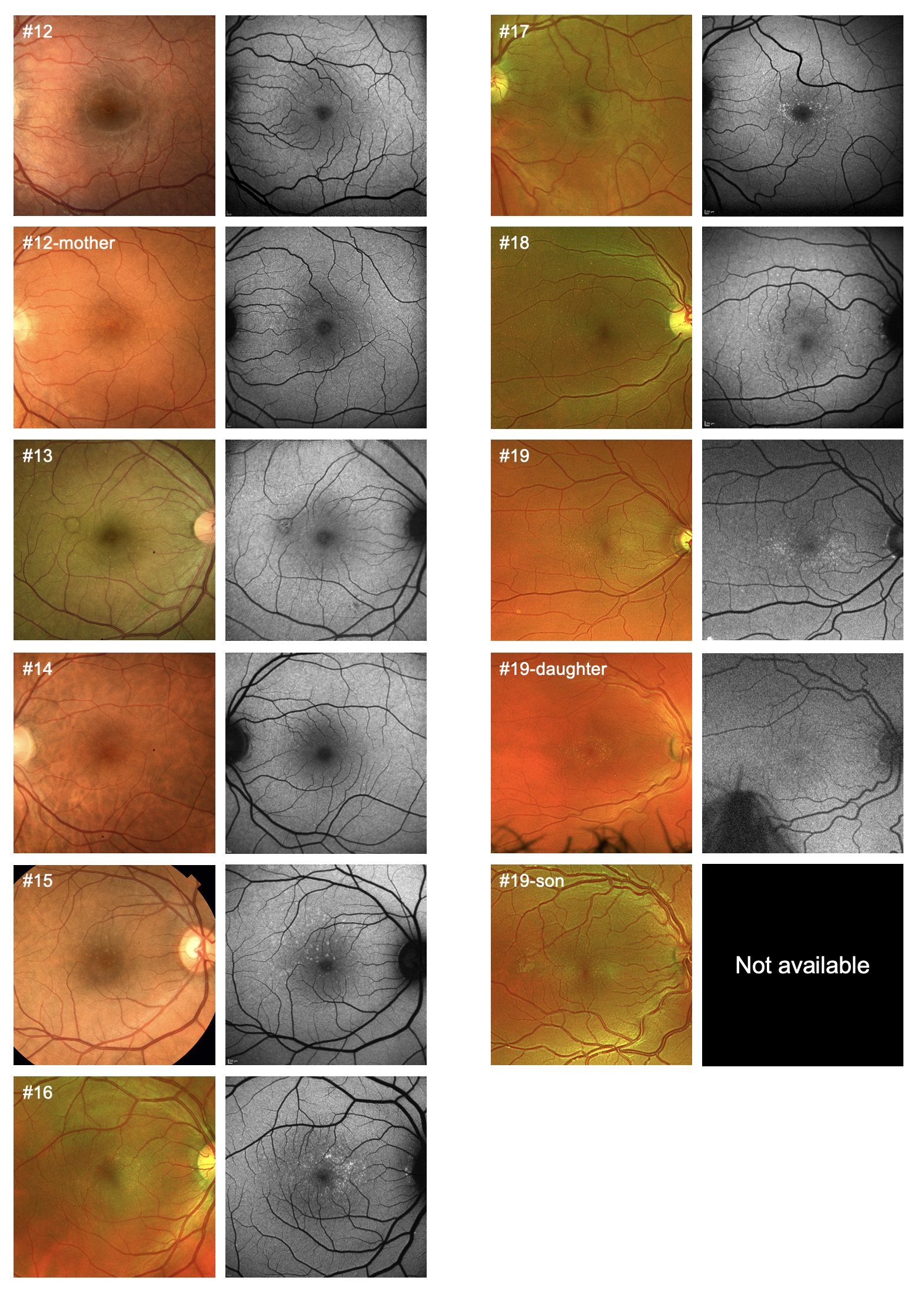

Supplement: Supplementary file 2 — Supplementary Figure 2 [file 41433_2024_3590_MOESM2_ESM.jpg]
